# Mechanisms in Growth-Promoting of Cucumber by the Endophytic Fungus *Chaetomium globosum* Strain ND35

**DOI:** 10.3390/jof8020180

**Published:** 2022-02-11

**Authors:** Yehan Tian, Xuesong Fu, Gongchen Zhang, Rui Zhang, Zhensheng Kang, Kexiang Gao, Kurt Mendgen

**Affiliations:** 1 Shandong Provincial Key Laboratory for Biology of Vegetable Diseases and Insect Pests, College of Plant Protection, Shandong Agricultural University, Taian 271018, China; tianyehan@163.com (Y.T.); fxs779421428@163.com (X.F.); ruizhangzrr@163.com (R.Z.); 2 Qingdao Academy of Agricultural Science, Qingdao 266100, China; gczhangnky@163.com; 3 State Key Laboratory of Crop Stress Biology for Arid Areas, College of Plant Protection, Northwest A&F University, Yangling 712100, China; kangzs@nwsuaf.edu.cn; 4 Department of Biology, University of Constance, 78457 Constance, Germany; kurt.w.mendgen@uni-konstanz.de

**Keywords:** endophytic fungi, *Chaetomium globosum*, cucumber, plant growth-promoting

## Abstract

Endophytic fungi are effective in plant growth and development by secreting various kinds of plant hormones and nutrients. However, the cellular and molecular interactions between the endophytic fungi and plant growth-promoting have remained less explored. The present study was designed to explore the effects of the infection and colonization events of *Chaetomium globosum* strain ND35 on cucumber growth and the expression pattern of some metabolically important genes in development of the cucumber radicle. The results demonstrated that strain ND35 can infect and colonize the outer layers (cortical cells) of cucumber root and form a symbiotic structure with the host cell, similar to a periarbuscular membrane and establish chemical communication with the plant. Through transcriptome analysis, we found the differentially expressed genes (DEGs) caused by strain ND35 were mainly enriched in phenylpropanoid biosynthesis, plant hormone signal transduction, plant-pathogen interaction and photosynthesis. Correspondingly, the contents of reactive oxygen species (ROS), hydrogen peroxide (H_2_O_2_), indole-3-acetic acid (IAA), gibberellin (GA), zeatin (ZT), salicylic acid (SA), jasmonic acid (JA) and the activity of phenylalanine ammonia lyase (PAL), 4-coumarate-CoA ligase (4CL), cinnamyl alcohol dehydrogenase (CAD), and peroxidase (POD) in ND35-colonized seedlings were generally higher than those of non-inoculated seedlings. Overall, the infection and colonization events of *C. globosum* strain ND35 increased cucumber growth through complex regulation of plant hormones biosynthesis and metabolism. Furthermore, although the endophytic fungus strain ND35 produced IAA, GA, ZT, and ergosterol in the fermentation broth, and there are enabled to promote growth of cucumber, it is uncertain whether there are ND35-derived microbial hormones in plants. This study of the interaction between cucumber and strain ND35 contributes to a better understanding of the plant-endophytic fungi interactions, and may help to develop new strategies for crop production.

## 1. Introduction

Healthy plants host a large and varied microbial community, the plant microbiota some confer fitness advantages to the plant host, including growth promotion, nutrient uptake, stress tolerance and resistance to pathogens [1]. Endophytes are important components of plant microbiomes, as microbes that live within plant tissues without causing symptoms of disease [2]. Endophytes usually come from the vertical transmission of host seeds and the horizontal transmission of air and soil microorganisms [3,4,5]. In the symbiotic relationship, the plant helps endophytes by providing nutrients and shelter, while endophytes can produce plant hormones, change antioxidant enzymes activity and promote growth of host plants under stress conditions [6,7]. It is demonstrated that endophytes can promote plant growth [8,9], enhance biomass production [3,10], and induce plant defense to the biotic and abiotic stresses, such as plant pathogens, drought and salinity [11,12,13,14]. In recent years, endophytes’ ability to assist in plant health, and as the biocontrol agent of plant diseases have been used to reduce the ecological and public health impacts caused by the overuse of chemicals and the emergence of fungicide resistance [2]. Endophytes are resources for plant production and biocontrol agents.

Endophytic fungi have been reported to increase plant growth and agricultural productivity [9,12]. The higher accumulation of primary and secondary metabolites in plant tissues in many plant-microbe interactions plays an important role in enabling plant growth and tolerance to abiotic and biotic stresses [13]. It has been reported that the interaction of *Cucumis melo* with *Trichoderma harzianum* affects growth of *Cucumis melo* through the systemic changes in the contents of several phytohormones, such as zeatin (ZT), indole-3-acetic acid (IAA), 1-aminocyclopropane-1-carboxylic acid (ACC), salicylic acid (SA) and jasmonic acid (JA) [15]. Endophytic fungi also significantly increased the productivity of pepper by modulating cell metabolic pathways including gibberellin (GA), auxin, and cytokinin (CTK) patterns [16]. Endophytic fungi isolated from common bean exhibited plant growth-promoting activities, including phosphate solubilization, ammonia production, nitrogen fixation, extracellular enzymatic activities, and IAA production [17]. Endophytic fungi associated with orchids can enhance the growth of orchid by synthesizing ammonia, which was easily taken up by host orchid [18]. Endophytic *Serendipita* spp. can increase the growth of *Arabidopsis* seedlings and plant biomass through the emission of volatile organic compounds (VOCs), such as methyl benzoate. The involvement of auxin and cytokinin signaling play important roles in *Serendipita* VOC-induced plant growth modulation [19]. The above mentioned studies demonstrated a strong change of hormonal and secondary metabolic pathways associated with growth stimulation in the plants following colonization by endophytic fungi.

*Chaetomium globosum*, one of the most common species of ascomycetes, possesses the ability to produce metabolites with bioactive potentials, such as chaetoglobosins, epipolythiodioxopiperazines, azaphilones, xanthones, anthraquinones, chromones, depsidones, terpenoids and steroids [20]. *C. globosum* exhibited a specific ability to control numerous foliar and root pathogens, including *Rhizoctonia solani*, *Pythium ultimum* and *Pyrenophora tritici-repentis* as a biocontrol agent [21]. The mechanisms by which *C. globosum* inhibits the pathogen growth are mainly based on the antibiosis in microbiology [21,22], while are on the activation of host defenses rather than antagonism in planta [23]. Chaetoglobosins produced by endophytic *C. globosum* displayed significant inhibition against the growth of *Sclerotinia sclerotiorum* mycelium [24]. Chaetoglobosins and their synergistic combinations with globosuxanthone, prenisatin showed tremendous potentiality in plant growth promotion of *Brassica* seedlings and high inhibitory action against *Sclerotium rolfsii* [25]. Meanwhile, Endophytic *C. globosum* has been reported to enhance copper stress tolerance in maize seedlings by regulating the osmotic solute content, antioxidant enzyme activity, and lipid peroxidation levels [26]. Endophytic *C. globosum* D38 significantly promoted the growth of *Salvia miltiorrhiza* seedlings and enhance the contents of both phenylpropionic acids and tanshinones in *S. miltiorrhiza* root by helping the host make better utilization of nutrients and enhance the primary metabolism fluxes [27]. *C. globosum* strain ND35 is a dominant endophytic fungus isolated from *Populus tomentosa* and display antagonistic activities against several common fruit and forest pathogenic fungi in vitro [21,28]. The strain ND35 also can be made into a biofertilizer for field crops and woody plant seedlings, which could colonize the plants, promote plant growth and effectively protect the host plant from the biotic and abiotic stresses [29,30,31,32,33]

A growth-promoting effect of strain ND35 could be observed during the whole lifetime of the plant, especially in cucumbers. When cucumber seedlings were inoculated with the strain ND35 and transferred to soil, the plants exhibited faster growth and earlier flowering. However, there is still much to learn about the processes by which strain ND35 infected and colonized cucumber roots, and the subsequent responses of cucumber. This study aimed to investigate the infection and colonization patterns of strain ND35 in cucumber radicles, and characterize the effects of strain ND35 on cucumber growth, as well as to identify candidate metabolic pathways involved in plant growth promotion.

## 2. Materials and Methods

### 2.1. Plant and Fungal Materials, Experimental Design

Cucumber (*Cucumis sativus* L. cv. ‘*Jinchun*’) seeds were purchased from the Xiangyun Seed Industry Co., Ltd. (Xintai, China). The endophytic *Chaetomium globosum* strain ND35 was preserved at Shandong Agricultural University. Ascospores of C. globosum strain ND35 were cultured on potato dextrose agar (PDA; per liter containing 200 g potato, 20 g glucose, 20 g agar) at 25 °C in the dark were collected by washing the plates with sterile distilled water and brought to a concentration of 1 × 10^8^ spores/mL.

An experiment was conducted to test the effect of different concentrations of strain ND35 spores on cucumber growth. Cucumber seeds, sterilized in 2% sodium hypochlorite for 5 min and washed in sterile distilled water, were germinated in Petri dishes containing sterile filter paper and 5 mL different concentrations of the ND35 strain spore suspension in the dark at 25 °C, the concentrations of the spore suspension were set to 0 spores/mL (CK), 1 × 10^4^ spores/mL (T1), 1 × 10^5^ spores/mL (T2), 1 × 10^6^ spores/mL (T3), 1 × 10^7^ spores/mL (T4), and 1 × 10^8^ spores/mL (T5). All seeds treatments were incubated in a dark growth chamber at 28 °C. The radicle length, fresh weight and dry weight of cucumber seeds were investigated at 12 h post inoculation (hpi). Each treatment contained three replicates, with 60 seeds per replicate. The germinated seeds for 36 h in the above treatment were transplanted into a seedling tray with seedling substrate and then kept in a climatic chamber (RLD-1000B-4, Ningbo Le Electrical Instrument Manufacturing Co., Ltd., Ningbo, China) at 28 °C, the relative humidity was 60 ± 5%, and the photoperiod was 14 h/10 h (light/dark). The 15-day-old seedlings with four to five leaves were used for testing the plant height, root length, shoot dry weight and root dry weight. All measurements were conducted on three replications per treatment, each replicate contained 15 seedlings.

An experiment was conducted to observe the infection and colonization of strain ND35 in the cucumber radicle and to test the effect of strain ND35 on the growth and development of cucumber. Cucumber seeds, sterilized in 2% sodium hypochlorite for 5 min and washed in sterile distilled water, were germinated in Petri dishes. Cucumber seeds placed in Petri dishes containing sterile filter paper with 5 mL strain ND35 spore suspension (1 × 10^6^ spores/mL) were used as the strain ND35 inoculation treatment (ND), while seeds placed in sterile filter paper with sterile water were taken as control (CK). The seedlings were grown in the dark at 25 °C. The cucumber radicles harvested at 12 hpi, 20 hpi, 24 hpi, 30 hpi, 36 hpi, 48 hpi, 60 hpi were used for further experiments, each treatment contained three replicates, each replicate contained 15 seeds. The further experiments included observation of the infection and colonization of strain ND35 in cucumber radicle, transcriptome analysis of cucumber radicle response to the infection and colonization of strain ND35, and the influence of strain ND35 on the endogenous hormonal and other secondary metabolites levels of cucumber.

An experiment was conducted to identify the secondary metabolites produced by strain ND35 and their roles in promoting cucumber growth, such as indole-3-acetic acid (IAA), indolebutyric acid (IBA), gibberellin (GA), zeatin (ZT) and ergosterol. The mycelia plugs of strain ND35 were inoculated in potato dextrose broth (PDB; per liter containing 200 g potato, 20 g glucose) for culture at 25 °C rotation speed at 200 rpm. After incubation for 15 days, fermented broth was harvested and then filtered with a sterile filter paper to remove the mycelia. The fermentation filtrate (1 L) of strain ND35 was adjusted to pH 3.8 (GA), 8.0 (ergosterol), 8.2 (ZT), 4.0 (IBA/IAA) with 1 M HCl solution or 1 M NaOH solution and extracted three times with 1 L portions of ethyl acetate. The solutions of ethyl acetate containing the secondary metabolites of strain ND35 were evaporated in a rotary evaporator (Shanghai Yarong Biochemistry Instrument Factory, Shanghai, China) and concentrated into yellowish-brown solid at 40 °C. Then, the yellowish-brown solid was dissolved in 5 mL of methanol for high performance liquid chromatography (HPLC) analysis. GA and ergosterol were separated by preparative reversed-phase high-performance liquid chromatography. The promoting effect of GA standard solution (3 μg/mL), ergosterol standard solution (3 μg/mL), GA crude product from strain ND35 (3 μg/mL), and ergosterol crude product from strain ND35 (3 μg/mL) on the cucumber growth was verified in the greenhouse.

### 2.2. Infection and Colonization of C. globosum ND35 on Cucumber Radicle in Vitro

Light microscopy (LM): The cucumber radicles were harvested at 12 hpi, 20 hpi and 24 hpi and stained for light microscopy using aqueous 0.05% toluidine blue in 1% sodium borate, and viewed on a Zeiss microscope (Zeiss, Jena, Germany). Interactions between the fungus and plant were photographed using a Zeiss Axioscope microscope (Zeiss, Jena, Germany) and a digital camera (Canon Power Shot G7, Tokyo, Japan). At least 10 samples were examined for each sampling time.

Scanning electron microscopy (SEM): Samples were collected 20, 24 and 30 h after ND35 spores inoculation and fixed for 12 h in 2% (*v/v*) glutaraldehyde in 0.1 M phosphate buffer (pH 7.2) at 4 °C, and then rinsed thoroughly for 6 times (30 min per time) with 0.1 M phosphate buffer (pH 7.2). Samples were post-fixed for 2 h in 1% (*v/v*) osmium acid in 0.1 M phosphate buffer (pH 7.2) at room temperature, rinsed thoroughly for 6 times (30 min per time), and then dehydrated in a graded acetone series (30, 50, 70, 80, 85, 90, 95 and 100%), each grade for 1 h and three times for 100% acetone. In addition, replaced in pure isoamyl acetate three times, each time for 30 min, subsequently dried in a critical point dryer (HCP-2, Hitachi, Tokyo, Japan), mounted on stubs, and then coated with gold (200 nm thickness) in a sputter coater (JFC-1600, JEOL, Tokyo, Japan). The coated specimens were examined and photographed with scanning electron microscopy (JSM-6610LV, JEOL, Tokyo, Japan) at 20 kV.

Immunofluorescence labeling: The preparation of pre-adsorption to remove antibodies recognizing unspecific epitopes and primary antibody was carried out according to the method of Ernst [3]. The mycelial powders used for the pre-adsorption were generated from *Botryosphaeria ribis* strains YL-JJ, *Rhizoctonia solani* strains Rs-1, R. solani strains Rs, *Pythium aphanidermatum* strains Pa, and *Fusarium oxysporium* strains F, which were prepared according to the method of Gao and Mendgen [4]. According to the amended method of Kang [34], immunofluorescence labeling was carried out as follows: after inoculation for 8 h and 12 h, samples were submerged in fixed transparent liquid (chloroform: dehydrated alcohol = 1: 4, and 0.15 g trichloroacetic acid added in 100 mL) for 12 h at 4 °C, then transferred to chloral hydrate for 1 h, washed twice with 50% dehydrated alcohol, 15 min per times, then rinsed 3 times with sterile demineralized water, blocked in 1% (*w/v*) bovine serum albumin in PBS (pH 7.2) for 30 min and washed 3 times with PBS, then incubated in primary antibody diluted 1:100 in PBS for 1 h, and rinsed 6 times with PBS, 5 min per times, then treated with goat anti-rabbit IgG-FITC (Solarbio, Beijing, China) diluted 1:10 in PBS for 1 h, and rinsed 6 times with PBS, 5 min per time, then washed for 1 min with sterile demineralized water, subsequently immersed in 50% dehydrated alcohol. Detected and photographed with a Nikon ECLIPSE 90i (Nikon, Tokyo, Japan) with fluorescence energizer and filter.

Resin Embedding and Transmission Electron Microscopy: The cucumber radicles at 36 hpi, 48 hpi and 60 hpi were treated using the method of Gao and Mendgen [4]. Root samples were fixed in 2.5% glutaraldehyde in 0.1 M cacodylate buffer at pH 7.4 with 1 mm CaCl and 1% (*w/v*) sucrose for 3 h at room temperature, and postfixed with 1% (*w/v*) osmium tetroxide in the same buffer for 2 h at room temperature. After being rinsed thoroughly with 0.1 M cacodylate buffer (pH 7.4), samples were dehydrated in an ascending series of ethanol. Fully dehydrated samples were moved from absolute ethanol through a 1: 1 mixture of ethanol and propylene oxide to pure propylene oxide. Samples were infiltrated through a series of Epon-Araldite-Mixture resin (Sigma-Aldrich, St. Louis, MO, USA) in propylene oxide, followed embedded in molds with fresh 100% resin and polymerized at 65oC for 36 h according to conventional protocols [4]. Semi-thin sections (3–5 μm) were cut on a Microtome (Leitz 1512, Wetzlar, Germany) using glass knives, stained for light microscopy using aqueous 0.05% toluidine blue O in 1% sodium borate, and viewed on a microscope. Images were captured using a Zeiss Axioscop microscope (Zeiss, Jena, Germany) and a digital camera (Canon Power Shot G7). Meanwhile, ultrathin sections of Epon-Araldit-Mixture embedded material were sectioned with glass knives on a Reichert microtome (Model OM-U3, Reichert-Jung (Leica), Germany), collected on formvar-coated copper grids, stained for 10 min in 2% uranyl acetate, and counter-stained for 5 min with 1.0% lead citrate. Sections were examined with a Zeiss EM 10 CR transmission electron microscope (TEM) (Zeiss, Oberkochen, Germany) at 60 kV. Three samples per sampling time were examined with an average of 10 grid squares per sample. At least 10 samples were examined for each species. More than three replicate experiments were performed.

### 2.3. Determination of Endogenous Metabolites and Enzymes in Cucumber Radicle

The cucumber radicles harvested at 24 hpi, 30 hpi, 36 hpi, 48 hpi, 60 hpi were used for the determination of the content (activity) of reactive oxygen species (ROS), hydrogen peroxide (H_2_O_2_), IAA, GA, ZT, SA, JA, lignin, phenylalanine ammonia lyase (PAL), 4-coumarate-CoA ligase (4CL), cinnamyl alcohol dehydrogenase (CAD) and peroxidase (POD) by enzyme-linked immunosorbent assay (ELISA) in Shanghai Jingmei Industrial Co., Ltd. (Shanghai, China). The content (activity) of ROS, H_2_O_2_, IAA, GA, ZT, SA, JA, PAL, 4CL, CAD and POD was calculated based on the OD values. 

### 2.4. Transcriptome Sequencing and Functional Analysis

The cucumber radicles harvested at 24 hpi, 30 hpi, 36 hpi, 48 hpi, 60 hpi were used for the mRNA sequencing. Total RNA was extracted from cucumber radicle using Trizol Reagent (Invitrogen, Carlsbad, CA, USA) and then evaluated using the NanoDrop ND-1000 spectrophotometer (ThermoFisher, Carlsbad, CA, USA). The sequencing libraries were generated using IlluminaTruSeq™ RNA Sample Preparation Kit (Illumina, San Diego, CA, USA). Briefly, mRNA was purified from total RNA using poly-T oligo-attached magnetic beads. Fragmentation was performed using divalent cations under an elevated temperature in an Illumina proprietary fragmentation buffer. First-strand cDNA was synthesized using random oligonucleotides and SuperScript II (Invitrogen, Carlsbad, CA, USA). cDNA synthesis was subsequently performed using DNA Polymerase I and RNase H. After adenylation of the 3′ ends of the DNA fragments, Illumina paired-end adapter oligonucleotides were ligated to prepare for hybridization. The library fragments were purified in an AMPure XP system (Beckman Coulter, Beverly, MA, USA) and sequenced by the Shanghai Personal Biotechnology Co. (Shanghai, China). To determine the gene expressions, the reference genome index was built using the Bowtie2 (2.2.6) software and the filtered reads were mapped to the reference genome of the *Cucumis sativus* L. var. *sativus* cv. 9930 (http://cucurbitgenomics.org/organism/2/, accessed on 20 October 2021) using the Tophat 2 (2.0.14) software based on improved burrows-wheeler transform (BWT) algorithm. The HTSeq (0.9.1) was used to determine the read counts of individually identified genes versus the expressions of these genes in the control libraries. FPKM (fragments per kilobase of exon model per million mapped reads) was used to standardize the expression levels, and DESeq (1.30.0) was used to analyze the differential expressions of genes. The cut-off value of *p* < 0.05 and at least 2 fold change were set as the threshold for differential expression.

### 2.5. Quantitative Real-Time PCR (qRT-PCR) Analysis

The cDNA used in RNA-seq was used for quantitative Real-Time PCR (qRT-PCR) analysis. Primers used for qRT-PCR were designed using the Primer Premier 5.0 software and listed in Supplementary Table S1. Quantitative PCR was performed using the SYBR Green qPCR kit on a Light Cycler^®^ 96 instrument (Roche Diagnostics, Mannheim, Germany). The relative expression levels of the analyzed genes were calculated using the 2^−^^△△CT^ method with 18S rRNA severing as an internal control [35].

### 2.6. Detection of Secondary Metabolites in Fermentation Broth of Strain ND35

The HPLC analysis was performed on an Agilent 1200 Series HPLC (Agilent Technologies, Santa Clara, CA, USA). The chromatographic column was Zorbax eclipse xdb-c18. All chromatographic analysis was performed at room temperature. The brand of the standard solutions of IBA, IAA, GA, ZT and ergosterol were Acros Organics part of Thermo Fisher Scientific (Waltham, MA, USA).

Detection of IBA, IAA and GA: The mobile phase consisted of methyl alcohol (A), water (B) and glacial acetic acid (C) (35:64:1, *v*/*v*/*v*): A, B, C, was used for the HPLC analysis with a flow rate of 1.0 mL/min. The detection wavelength for the IBA, IAA and GA was set at 254 nm, 280 nm and 254 nm, respectively, and 20 μL samples were injected.

Detection of ZT: The mobile phase consisted of A, B and C solutions (45: 51: 4, *v*/*v*/*v*) was used for the HPLC analysis with a flow rate of 1.0 mL/min. The detection wavelength was set at 280 nm and 10 μL samples were injected.

Detection of ergosterol: The mobile phase consisting of A and B solutions (95:5, *v*/*v*) was used for the HPLC analysis with a flow rate of 0.7 mL/min. The detection wavelength was set at 280 nm and, 20 μL samples were injected.

### 2.7. Statistical Analysis 

All numerical data collected from this study were subjected to statistical analysis and significance tests. Specifically, cucumber physiological data were analyzed by one-way analysis of variance (ANOVA), and statistical significance was determined by pairwise comparisons using Student-Newman-Keuls (SNK) test or Student’s *t*-test (*p* < 0.05), which was implemented in SPSS 22.0 (IBM, Armonk, NY, USA).

## 3. Results

### 3.1. Effect of C. globosum Strain ND35 on the Cucumber Growth

**Figure 1:**
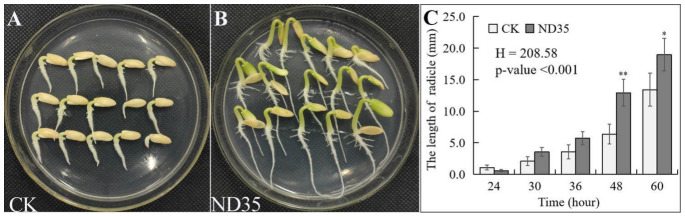
Effect of *C. globosum* strain ND35 on the radicle length of cucumber. (**A**): The radicle length of cucumber treated without strain ND35 at 48 h; (**B**): The radicle length of cucumber treated with strain ND35 at 48 h; (**C**): The time course of radicle length in different treatment. The spore concentration of *C. globosum* strain ND35 was 1 × 10^6^ spores/mL. * indicates significant differences at *p* < 0.05, ** indicates significant differences at *p* < 0.01 (by *t*-test), the below is same.

The effect of strain ND35 treatment on the germination of seed and growth of cucumber seedlings varied with different concentrations of spores. As shown in Table 1, the cucumber seeds inoculated with 1 × 10^4^, 1 × 10^5^, 1 × 10^6^ spores/mL ND35 suspension presented higher seed radicle length, fresh weight, and dry weight after 12 h germination, and also higher plant height, root length, shoot dry weight, and root dry weight of seedlings with 15-days-old than those of non-inoculated groups. However, higher concentration inoculation of strain ND35 spore suspension (1 × 10^7^, 1 × 10^8^ spores/mL) inhibited the growth of cucumber as compared with lower concentration inoculation. The strain ND35 at the concentration of 1 × 10^6^ spores/mL exhibited the best growth-promoting effect on cucumber. The radicle length, fresh weight of radicle, dry weight of radicle, plant height, shoot dry weight, and root dry weight of cucumber inoculated with strain ND35 at the concentration of 1 × 10^6^ spores/mL were significantly increased by 66.9%, 449.1%, 239.5%, 14.1%, 16.7%, and 68.1% compared to those of non-inoculated groups, respectively. Interestingly, the growth vigor of seeds on the seedbed soaked with strain ND35 spore suspension (1 × 10^6^ spores/mL) was lower than that in non-inoculated groups in the first 24 h, while exhibited significantly higher growth rate after 48 h compared with the control (Figure 1).

### 3.2. Secondary Metabolites of C. globosum Strain ND35 

In cultural solutions extracted from strain ND35, we detected large amounts of indolebutyric acid (IBA), indole-3-acetic acid (IAA), gibberellin (GA), zeatin (ZT) and ergosterol by HPLC (Supplementary Figure S1A–E). The extracts of IBA, IAA, GA, ZT, and ergosterol in the fermentation broth of strain ND35 reached 12.54 mg/L, 13.70 mg/L, 0.78 mg/L, 0.03 mg/L and 14.75 mg/L, respectively (Supplementary Table S2). Using the preparative reversed-phase high-performance liquid chromatography, we obtained the crude product of GA and ergosterol from the fermentation broth of strain ND35, and further evaluated the effects of the crude products of GA (GA-T) and ergosterol (Er-T) on cucumber growth. The plant height, root length, fresh weight, and dry weight of cucumber seedlings were significantly increased by 48.19%, 28.99%, 55.01% and 29.12% under GA-T treatment and by 31.40%, 30.79%, 53.37% and 28.92% under Er-T treatment, respectively (Supplementary Figure S1F–I). Meanwhile, the standard solution of GA (GA-S) and ergosterol (Er-S) significantly increased the plant height, root length, fresh weight, and dry weight compared to the control treatment (CK), while the Er-S samples and control treatment displayed no significant differences in terms of fresh weight.

### 3.3. Infection and Colonization of C. globosum Strain ND35 Spores on the Radicle of Cucumber Seeds

**Figure 2:**
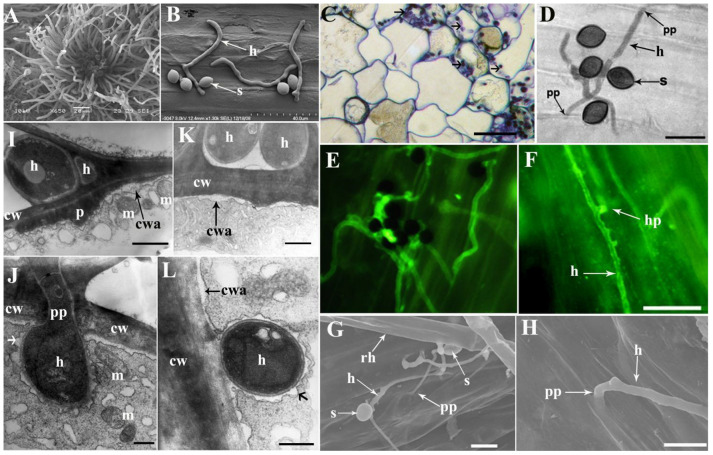
Infection and colonization of *C. globosum* strain ND35 in cucumber radicle. Under scanning electron microscopy, (**A**): The morphology of appendage of strain ND35 perithecium; (**B**): The morphology of hyphae from germinated ascospores of strain ND35. C-L: Colonization of hyphae of strain ND35 in cucumber radicle. Under light microscopy, (**C**): Several epidermal cells were infected by hyphae (arrowheads) of strain ND35, bars = 10 μm; (**D**): The hyphae of strain ND35 invaded cucumber root from a gap between epidermal cells of the root surface at 20 hpi (arrowheads), bars = 12 μm. (**E**–**H**): The hyphae developed from germinated germ tube penetrated the radicle from a gap between the epidermal cells at 20 hpi; (**E**) and (**F**): The hyphae of strain ND35 stained with goat anti-rabbit conjugated FITC and viewed with a fluorescence microscope, bars = 20 μm. Under scanning electron microscopy, (**G**): The hyphae of strain ND35 penetrated the cucumber from a gap between epidermal cells at 20 hpi, bars = 10 μm; (**H**): The hypha penetrated the epidermis from a gap between epidermal cells at 30 hpi, bars = 5 μm. (**I**–**L**): TEM imaging of defenses reaction of cucumber radicle cells at contacting or penetration sites and hyphae in outer cortical cells; (**I**): Papilla was formed in the inner wall of the epidermal cell when hyphae of strain ND35 between epidermal cells have contacted the outer wall of epidermal cells at 36 hpi, bars = 1 μm; (**J**): The hypha of strain ND35 penetrated the cell wall into the adjacent cortical cell at 36 hpi, bars = 0.5 μm; (**K**): Cell wall apposition was formed on adjacent cortical cell wall when hyphae have been growing in intercellular space contacting cell wall, bars = 0.5 μm; L: The hypha of strain ND35 in cortical cells were surrounded by the amorphous matrix (arrowheads), bars = 0.5 μm. cw: cell wall, cwa: cell wall apposition, m: mitochondrion, h: hypha, hp: hyphopodia, p: papilla, pp: penetration point, rh: root hair, s: spores of strain ND35.

*C. globosum* strain ND35 is an ascomycete fungus, which generates spores in perithecium (Figure 2A). The ascospore of strain ND35 was lemon-shaped, and the germ tube only emerged at one side of the ascospore (Figure 2B). The infection of strain ND35 on the cucumber started with deposition and adhesion of ascospores on the surface of the radicle. Under the light microscopy, a large number of germinated ascospores were found on the roots, especially on the root hair zone after inoculation for 16–24 h. Several epidermal cells started to be infected by hyphae of strain ND35 at 24 h post inoculation (hpi). Epidermal and outer cortical cells were infected and colonized by hyphae of strain ND35 at 24–72 hpi (Figure 2C). Hyphae were present within root hairs, epidermal cells, and cortical cells. The hypha developed from the germ tube invaded the cucumber tissue at the gap between epidermal cells of the root surface (Figure 2D) or directly penetrated the surface tissue. The hyphae labeled with goat anti-rabbit IgG conjugated fluorescein isothiocyanate (FITC) appeared green. The hyphae of strain ND35 pushed epidermal cells apart and squeezed through the cleft that may have resulted from hyphal tip pressure and invaded into root at 20 hpi (Figure 2E,F).

A more detailed picture of the development of hyphae from germinated ascospore on the root surface and initiation of penetration structures was obtained by SEM experiments. After inoculation for 8–12 h, the hyphae abundantly adhered to the surface of roots, where they had settled in the grooves guiding the hyphal growth along the root cell direction (Figure 2G). Extensive colonization by a dense network of hyphae was observed on the surface of the root. The hyphae also grew and aligned to main and lateral root surfaces, as well as to root hairs. Hyphae were especially dense in cracks and compartments between root cells, as well as injunctions of lateral roots with the main root. Here, they established an intimate contact with the epidermis. The hypha developed from the germ tube invaded the cucumber tissue from the gap between epidermal surface cells (Figure 2H). Hyphae within epidermal cells were observed under transmission electron microscopy (TEM) after inoculation with strain ND35 for 24 h. Epidermal cell wall-bound phenolics accumulated and saponified at sites of attempted fungal ingress, forming cell wall apposition (CWA) and papilla, when hyphae of strain ND35 in an epidermal cell contacted adjacent outer cortical cell at 36 and 48 hpi (Figure 2I,J). In the contact region, the constrictive hyphae penetrated through the cell walls and entered the cortical cells. TEM images showed that after penetration, the invasive hyphal apex swells to form a metamorphosis hyphae, and the penetration peg acted as a hyphal neck. The expanded growth of the hyphal apex leads to the invagination of the host plasma membrane and creates an interface between the host and fungal hyphae. The fungal hyphae in cortical cells were surrounded by the plant-derived plasma membrane, similar to the periarbuscular membrane (PAM) [36] which is a symbiotic structure of arbuscular mycorrhiza (AM) and plants (Figure 2J–L). Meanwhile, cytoplasmic aggregates, which hold mitochondrion and vesicles, accumulated beneath the cell wall apposition and papilla, presumably providing the energy and precursors necessary for CWA and papilla formation (Figure 2I,J).

### 3.4. Transcriptome Sequencing and Functional Analysis of Differentially Expressed Genes (DEGs)

**Figure 3:**
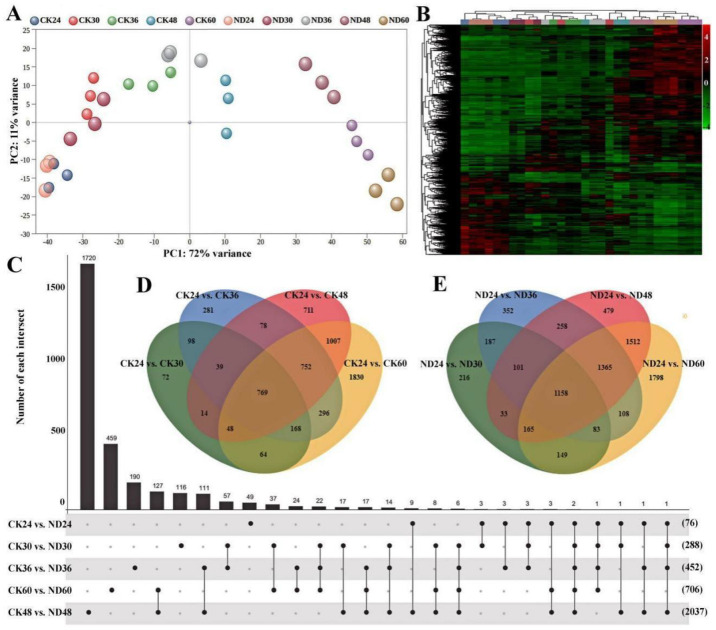
Gene expression profiling and the number of differentially expressed genes (DEGs) analysis in responding to the infection and colonization of *C. globosum* strain ND35 in cucumber radicle. (**A**): Principal Component Analysis (PCA) plots showing the differences between samples according to the genes expression; (**B**): Hierarchical clustering analysis showing the expression patterns of differentially expressed genes (DEGs) in different treatments, the color represents the expression level of differential genes (log2), the red represents the higher expression levels genes, and the green represents lower expression levels genes; (**C**–**E**): Venn diagrams showing overlap of DEGs between different treatments.

Preliminary transcriptome analyses were conducted to investigate the impact of infection and colonization of *C. globosum* strain ND35 on gene expression in cucumber seedlings. A total of 30 libraries (three biological replicates, with or without strain ND35) were sequenced, resulting in a total of 37.36 to 73.86 million clean reads per sample, the Q30 in each sample was above 93% (Supplementary Table S3). High-quality reads were mapped to the *Cucumis sativus* L. var. *sativus* cv. 9930 reference genome [37]. Overall, 96% of the clean reads mapped to the reference genome. In total, 15004–16644 genes were found to be expressed per sample. In general, good correspondence and correlation correlations (0.90–0.99) were found between the biological replicates (Supplementary Figure S2). PCA showed a distinguishable distribution between the sample treated with strain ND35 and non-treated control (Figure 3A), and gene expression clusters of group CK24 were close to group ND24. With seedlings’ development, the gene expression clusters of group CK36, CK48, and CK60 were clearly departed from group ND36, ND48 and ND60, respectively. Similar results were also detected in the heatmap (Figure 3B). Specifically, 45, 242, 332, 1445, 444 up-regulated genes and 31, 46, 120, 592, 262 down-regulated genes were measured in ND24, ND30, ND36, ND48 and ND60 treatment groups compared with CK24, CK30, CK36, CK48, and CK60, respectively (Figure 3C and Supplementary Table S4). In the control group without strain ND35 treatment, 1272, 2481, 3418, and 4934 differentially expressed genes (DEGs) were detected in the CK30, CK36, CK48, and CK60, respectively, compared with CK24 (Figure 3D). However, in the processing group treated with strain ND35, 2092, 3612, 5071, and 6338 DEGs were detected in the ND30, ND36, ND48, and ND60, respectively, compared with ND24 (Figure 3E).

**Figure 4:**
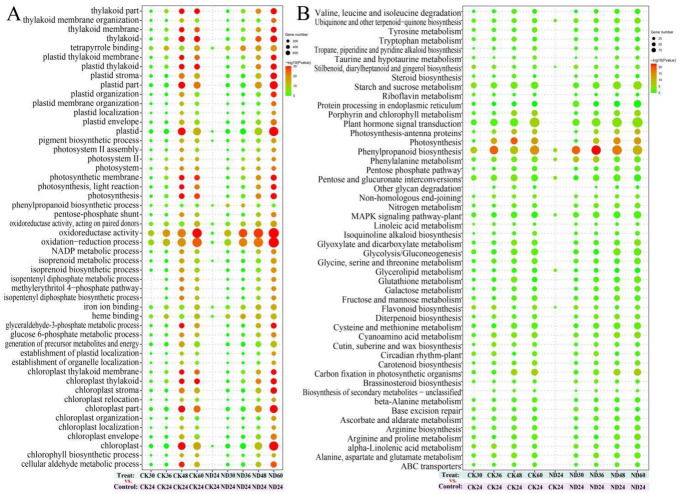
GO and KEGG enrichment analysis in responding to the infection and colonization of *C. globosum* strain ND35 in cucumber radicle. (**A**): Gene Ontology (GO) enrichment analysis; (**B**): Kyoto Encyclopedia of Genes and Genomes (KEGG) enrichment analysis. DEGs were classified into specific biological process categories and pathway categories with high classification stringency (*p* < 0.05). The circle size represents the number of genes in the category. The circle color represents the significance probability, red represents higher significance levels, green represents lower significance levels.

Gene Ontology (GO) and KEGG pathway enrichment analyses were conducted to unravel the major functions of the DEGs. As the seed germinating time prolongs, a total of 19 major GO terms, such as oxidation-reduction process, oxidoreductase activity, plastid, chloroplast, and thylakoid were found to enrich in the DEGs of CK30 vs. CK24, CK36 vs. CK24, CK48 vs. CK24, and CK60 vs. CK24 (Figure 4A). The number of DEGs related to these transcripts increased at the late germinating stage relative to the previous time point, implying that these genes probably played potential roles in cucumber development. Meanwhile, the up-regulation of the genes related to oxidation-reduction process, oxidoreductase activity, phenylpropanoid biosynthetic process, isoprenoid metabolic process, pigment biosynthetic process, plastid, tetrapyrrole binding, chloroplast, heme binding, and iron ion binding were found in the groups treated with strain ND35 in comparison to the non-treatment control (Figure 4A and Supplementary Table S5). Eight of the GO terms enriched in the DEGs of the group CK24 vs. ND24, namely cell wall, cell wall modification, cell wall organization, cell wall organization or biogenesis, external encapsulating structure, external encapsulating structure organization, galacturonan metabolic process, and structural constituent of the cell wall (Supplementary Figure S3), implying that these genes probably played potential roles in cucumber response to infection and colonization of *C. globosum* strain ND35, providing further support to the results reported in the previous section.

**Figure 5:**
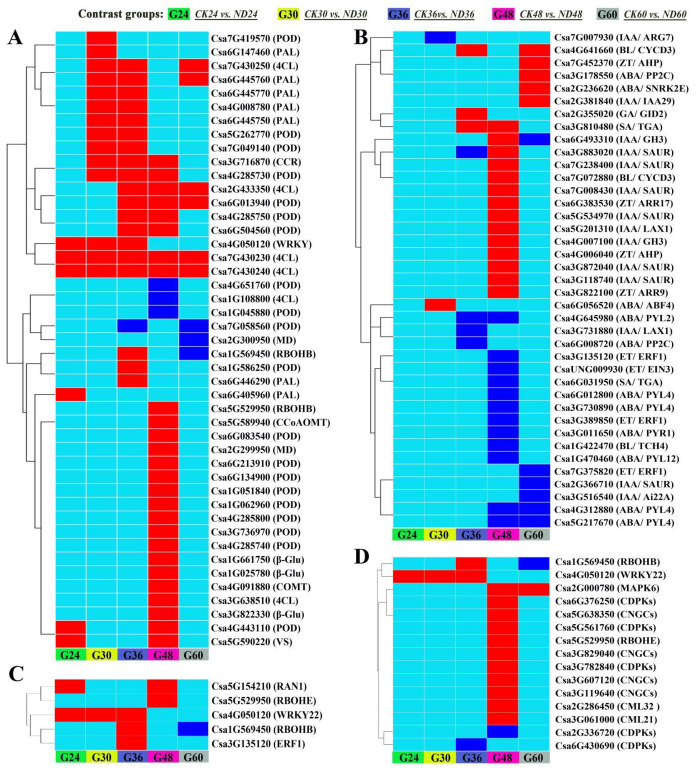
Gene expression patterns of major metabolic pathways in responding to the infection and colonization of *C. globosum* strain ND35 in cucumber radicle. (**A**): Phenylpropanoid biosynthesis; (**B**): Plant hormone signal transduction; (**C**): MAPK signaling pathway-plant; (**D**): Plant-pathogen interaction. The data point color indicates up-regulation (red), down-regulation (blue), and non-regulation (light blue) that is within the specified contrast group.

KEGG pathway enrichment analysis revealed that the genes related to phenylpropanoid biosynthesis, phenylalanine metabolism, plant hormone signal transduction, carbon fixation in photosynthetic organisms, brassinosteroid biosynthesis, photosynthesis, and so on were enriched in DEGs of CK30 vs. CK24, CK36 vs. CK24, CK48 vs. CK24, CK60 vs. CK24, during cucumber growth (Figure 4B and Supplementary Table S6). Meanwhile, the number of DEGs related to these transcripts increased in the groups treated with strain ND35 in comparison to the non-treatment groups. Phenylpropanoid biosynthesis genes were enriched in DEGs of CK24 vs. ND24, CK30 vs. ND30, CK36 vs. ND36, CK48 vs. ND48, and CK60 vs. ND60, these genes included β-glucosidases, phenylalanine ammonia lyases (PAL), 4-coumarate-CoA ligases (4CL), cinnamoyl-CoA reductases (CCR), caffeic acid 3-O-methyltransferases (COMT), caffeoyl-CoA O-methyltransferases (CCoAOMT), and peroxidases (POD) which might be involved in cell wall modification or mechanical stress response (Figure 5A). Plant hormone signal transduction genes were mainly enriched in DEGs of CK36 vs. ND36, CK48 vs. ND48, and CK60 vs. ND60, these genes were involved in the synthesis of gibberellin, auxin, jasmonic acid, zeatin, lignin, salicylic acid, and abscisic acid that might regulate growth and development of cucumber radicle (Figure 5B). The indole-3-acetic acid-amido synthetase genes (IAA/GH3: Csa4G007100, Csa6G493310), auxin-responsive protein genes (IAA/IAA29: Csa2G381840), auxin transporter-like protein genes (IAA/LAX1: Csa5G201310), auxin-induced protein genes (IAA/SAUR: Csa3G118740, Csa3G872040, Csa3G883020, Csa5G534970, Csa7G008430, Csa7G238400), histidine-containing phosphotransfer protein genes (ZT/AHP: Csa4G006040, Csa7G452370), two-component response regulator (ZT/ARR: Csa6G383530, Csa3G822100), and cyclin-D3 (BL/CYD3: Csa4G641660, Csa7G072880) were clearly up-regulated in ND48 compared to those of CK48. While the abscisic acid receptor genes (ABA/PYL: Csa1G470460, Csa3G011650, Csa3G730890, Csa4G312880, Csa4G645980, Csa5G217670, Csa6G012800) were clearly down-regulated in ND48 compared to those of CK48. These results indicate that the biosynthesis of endogenous phytohormones might be modulated by the strain ND35 in cucumber, such as IAA, GA, CTK, and abscisic acid (ABA). Furthermore, some DEGs involved in the plant-microbe interactions were identified in the cucumber plant after *C. globosum* strain ND35 infection and colonization (Figure 5C,D). Most of them were involved in the Ca^2+^ and MAPK signaling pathways and were significantly up-regulated at 48 hpi. The calcium-binding protein genes (CML: Csa2G286450, Csa3G061000), cyclic nucleotide gated channel (CNGC: Csa3G119640, Csa3G607120, Csa3G829040, Csa5G638350), calcium-dependent protein kinase (CDPK: Csa3G782840, Csa5G561760, Csa6G376250), MAPK (Csa2G000780) and respiratory burst oxidase (Rboh: Csa1G569450, Csa5G529950) were up-regulated in ND48 compared to those of CK48, which indicates that the response of cucumber to strain ND35 mainly referred to the signal transduction upon CML/CNGC/CDPK and the activation of response genes and the outbreak of ROS. Additionally, genes related to the cell wall reinforcement were also induced in ND24, ND30 and ND36, such as WRKY transcription factor 22 (WRKY22, Csa4G050120) and pectinesterase (Csa1G538170, Csa3G126810, Csa5G167190) (Supplementary Table S7). All the results indicated that the defense response of cucumber was induced by strain ND35 infection, such as the production of ROS, cell wall reinforcement and the expression of defense response genes (Figure 5C,D).

Moreover, qRT-PCR was carried out to validate the relative expressions of phenylpropanoid biosynthesis and plant hormone signal transduction transcripts, of which 29 highly expressed genes in cucumber radical presented consistent trends with RNA-seq results during the seed germination and seedling growth (Supplementary Figure S4). Taken together, genes related to phenylpropanoid biosynthesis and plant hormone signal transduction were obviously enriched in the groups treated with strain ND35 relative to the groups treated without strain ND35.

### 3.5. Influence of C. globosum Strain ND35 on the Endogenous Hormonal and Other Secondary Metabolites Levels of Cucumber

**Figure 6:**
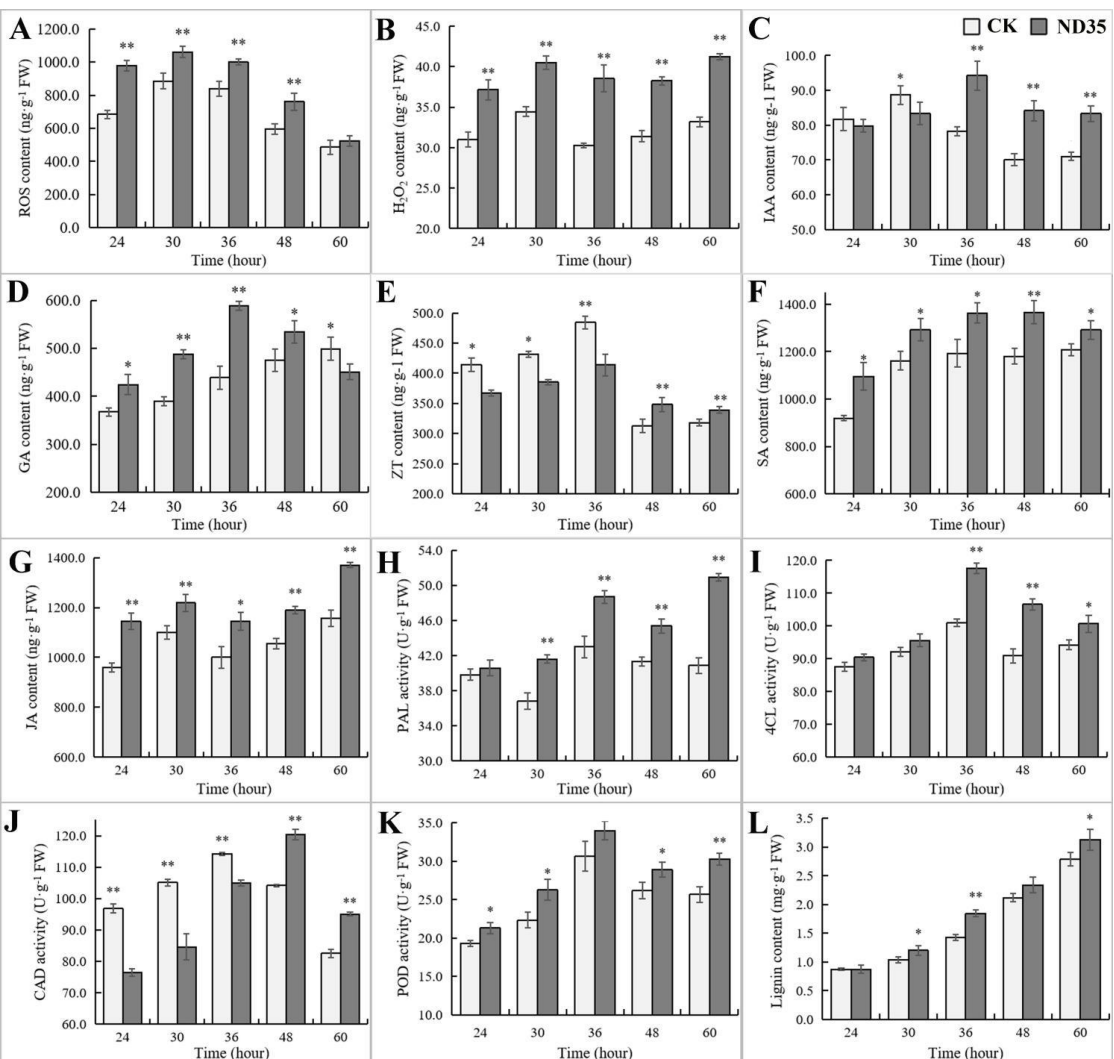
The changes of the biologically active substance of cucumber in responding to the infection and colonization of *C. globosum* strain ND35 in cucumber radicle. (**A**): The content of reactive oxygen species (ROS); (**B**): The content of hydrogen peroxide (H_2_O_2_); (**C**): The content of indole-3-acetic acid (IAA); (**D**): The content of gibberellic acid (GA); (**E**): The content of zeatin (ZT); (**F**): The content of salicylic acid (SA); (**G**): The content of jasmonic acid (JA); (**H**): The activity of phenylalanine ammonia lyase (PAL); (**I**): The activity of 4-coumarate-CoA ligase (4CL); (**J**): The activity of cinnamyl alcohol dehydrogenase (CAD); (**K**): The activity of peroxidase (POD); (**L**): The content of lignin. * indicated that there are statistically significant differences between groups based on Student’s t-test (*p* < 0.05). ** indicated that there are statistically significant differences between groups based on Student’s t-test (*p* < 0.01).

In agreement with the results of transcriptome sequencing, infection and colonization of *C. globosum* strain ND35 induced a series of changes in plant secondary metabolites, especially in plant endogenous hormones and lignin biosynthesis. During the germination of cucumber seeds, reactive oxygen species (ROS) content rose at the early germination stage (24–30 h), while gradually declining at the late stage from 36 to 60 h (Figure 6A). The hydrogen peroxide (H_2_O_2_) content of germinated seeds was relatively stable (Figure 6B), and the infection and colonization of *C. globosum* strain ND35 significantly increased the content of ROS and H_2_O_2_ in the germinated seeds compared with the non-inoculation control. The IAA levels showed the first rise and then decline trends (Figure 6C). IAA levels in strain ND35-colonized seedlings was significantly increased at the late germination stage (36–60 h). At the early germination stage (24–48 h), the GA content distinctly rose in germinated seeds treated with strain ND35 compared to that of non-treated control (Figure 6D). The content of zeatin (ZT) in strain ND35-colonized seedlings was low at the germination stage from 24 to 36 h and significantly increased at the late germination stage from 48 to 60 h compared to that of non-treated control (Figure 6E). Metabolites related to the biosynthesis of JA and SA showed obvious change after strain ND35 colonization, the content of SA and JA in ND35-colonized seedlings were significantly higher than those of non-treated control (Figure 6F,G). Meanwhile, key enzymes of lignin biosynthesis, such as phenylalanine ammonia lyase (PAL), 4-coumarate-CoA ligase (4CL), cinnamyl alcohol dehydrogenase (CAD), and peroxidase (POD), also showed distinct changes after strain ND35 colonization, these activity in samples treated with strain ND35 were significantly higher than those of samples non-treated with strain ND35 (Figure 6H–K), except, the activity of CAD was inhibited by strain ND35 at the early germination stage (24–36 h). During the seed germination, the lignin content of the radicle increased continuously as the seed germinated, strain ND35 inoculation obviously promoted the accumulation of lignin in the radicle (Figure 6L).

## 4. Discussion

Endophytes are common in plants, and many studies have shown that endophytes can stimulate plant growth or increase natural resistance in host plants [38,39,40]. Our results demonstrated that endophytic *C. globosum* strain ND35 can promote the growth of the cucumber plant in terms of radicle length, plant height, root length, fresh weight and dry weight, through efficient colonization in the cucumber root system and enhancing the biological process of phytohormone homeostasis, antioxidant activity, phenylpropanoid biosynthesis. These findings are consistent with the previous studies that a direct way of endophytic fungi promoted plant growth through producing phytohormones, especially gibberellins and IAA [41,42]. It has been reported that the endophytic association of *Aspergillus fumigatus* significantly improves plant growth characteristics by producing gibberellins [42]. In addition, fungal endophytic metabolites also can increase the plant biomass and yield traits [17]. For instance, higher ergosterol concentrations usually resulted in increased plant growth and development by up-regulating the expression of genes involved in the JA/ET signaling pathways in tomato plants [43,44]. Although it has been reported that the microbial secondary metabolites have been proved highly beneficial to plant growth, the changes in host phenotype may be more dependent on their own secondary metabolite induced by endophytic fungi or microbial metabolites. 

During the interaction between plants and microorganisms, plants employ a complex of physical and chemical defense strategies to respond to attempted invasions, especially pathogens. In general, endophytic fungi invade host cells without triggering obvious structural defense reactions, while hyphae of arbuscular mycorrhizal fungi may induce slight changes in cell walls. When endophytic fungi form intercellular hyphae or intracellular hyphal coils or arbuscular, the host plasma membrane invaginates and proliferates around all the developing fungal structures, forming an apoplastic space bordered by a specialized symbiotic interface [45,46,47]. This is one of the most important events in the successful colonization of plant cells by endomycorrhizal fungi, providing the structural basis of the biotrophic interaction between plant and fungus. The symbiont of AM mycorrhiza formed by arbuscular mycorrhizal fungi and host root represents an important paradigm for plant biotrophic interactions. The arbuscular mycorrhizal fungus colonizes the host cortex and differentiates within the cortical cells developing highly branched hyphae called arbusculars [48]. Each arbuscular within cortical cells is surrounded by a plant-derived periarbuscular membrane that is continuous with the plant plasma membrane or interfacial matrix material and excludes the fungus from the plant cytoplasm [33]. In this study, the infection and colonization events of strain ND35 included hyphae pushing root epidermal cells into cortical cell gap and penetrating cell wall into cortical cells, a combination of mechanical forces and enzymatic activities may contribute to the success of colonization of strain ND35. This mode of penetration was very similar to that reported for hyphae of *Stagonospora* sp. on or in the reed root [4]. After penetration, the hyphal apex expanded in the cortical cell and formed functional hyphae that were surrounded by the plant-derived plasma membrane, similarly to the plant-derived periarbuscular membrane.

From a cell biology perspective, intracellular accommodation of endosymbiont was a feat of coordinated cellular rearrangements, including cytoskeletal alterations, constriction of the central vacuole, endoreduplication, increased cytoplasm and plasma membrane [48]. In the symbiotic system of cucumber and *C. globosum* strain ND35, intracellular accommodation of cucumber by strain ND35 was observed, such as the accumulation of mitochondrion and vesicles, the formation of cell wall appositions (CWAs) or papillae, and the formation of the symbiotic interface, which was a common developmental program for the symbiosis of endophytic fungus hypha and host plant. It has been clarified that plants’ symbiosis signaling pathway, downstream transcriptional regulators, metabolic changes and nutrient transport were involved in the response of plants to endophytes symbiosis [49,50]. The response of plants to endophytes symbiosis may lead to better fitness of the host to the colonization of endophytes. As was reported in the interaction between tomato and *Trichoderma harzianum*, tomato root modulates its transcriptional machinery to further promote positive interaction with *T. harzianum* [51]. *T. harzianum* triggered the upregulation of genes related to ROS detoxification, auxin signaling, cell wall strengthening and redox balance. Transcriptome analyses identified a total of 984 DEGs in tomatoes that interacted with *T. afroharzianum*, which were mainly engaged in the biological process of phytohormone homeostasis, antioxidant activity, phenylpropanoid biosynthesis and glutathione metabolism [52]. Consistent with previous findings, expression of active oxygen, phytohormone biosynthesis and phenylpropanoid biosynthesis related genes were systemically activated in cucumber during early or long-term interaction with strain ND35, which may take part in the primed response of cucumber to the infection and colonization of strain ND35, and the cucumber growth promotion.

Calcium (Ca^2+^) is a universal second messenger involved in various cellular processes and has been considered a crucial component in modulating plant development and biotic and abiotic stress responses [53,54]. Our results also proved that the genes of Ca^2+^-binding proteins involved in the response of cucumber to strain ND35, including CaM, CML, CDPK, CNGCs, WRKY22, and respiratory burst oxidase homolog protein (RBOH), which indicates that the early response of cucumber to the colonization of strain ND35 mainly referred to Ca^2+^ signal transduction and the outbreak of ROS. Previous studies have revealed a sustained and rhythmic nuclear Ca^2+^ oscillations play a central role in tip growth of root hairs, endophytes symbiosis and biotic stress [55]. In addition, ROS interact with Ca^2+^ to propagate a cell-to-cell signal as an important second messenger [56]. In *Arabidopsis*, hyperpolarization-activated Ca^2+^ channels (HACCs) mediates Ca^2+^ influx dominates in root hairs, the activity of HACC was activated by NADPH oxidase respiratory burst oxidase homolog C (RBOHC) or reactive oxygen species (ROS) accumulation [57]. Alterations in intracellular Ca^2+^ concentration are sensed by Ca^2+^-binding proteins, including calmodulin-like proteins (CMLs), Ca^2+^-dependent protein kinases (CDPKs), calcineurin B-like proteins (CBLs) and cyclic nucleotide gated channel (CNGCs), CNGC members as central regulators of Ca^2+^ oscillations [58,59,60]. The defense response of cucumber to *Meloidogyne incognita* mainly referred to signal transduction upon CaM, CML, CDPK, and CNGCs [61].

Both mutualistic and biotrophic pathogenic fungi rely on living host plants for growth and reproduction and must modify host cell structure and function for successful infection [62]. The plant cell wall is a strong fibrillar network that provided a passive structural barrier to prevent the invasion of fungal microorganisms. In addition, the cell wall appositions (CWAs) or papillae function in hindering the entry of fungal pathogens into host cells by providing a structural collar [63]. Phenolic polymers such as lignin are components of the plant cell wall and structural barriers such as CWAs, which play fundamental roles in the biotic and abiotic interactions [64]. Phenylpropanoid metabolism plays a very important function in the biosynthesis of cell wall components [65,66]. During the development of cucumber seedlings, the expression of many genes related to the phenylpropane pathway was gradually up-regulated in cucumber radicle treated with strain ND35, thus lignin content and the activity of PAL, 4CL, CAD, and POD were increased in the seedlings. It seems that the presence of strain ND35 strengthened host cell walls by the formation of CWAs or papillae, the formation of CWAs or papillae may be attributed to the enhancement of phenylpropane metabolic activity (Figure 2–6). Many relevant studies have proved that microorganisms promote plant growth by enhancing phenylpropane metabolisms, such as *Bacillus pumilus* LZP02 promoted the growth of rice roots by enhancing carbohydrate metabolism and phenylpropanoid biosynthesis [67].

Endophytic fungi might manipulate the surveillance system of cell wall integrity for the establishment of a symbiotic system, to establish an association with downstream transcriptional regulators, such as plant defense signaling pathways and phytohormone signal pathways [63,68]. It has been suggested that cell cycle reactivation during the colonization of AM fungi triggers cell division in some cortical cells [68]. Changes in levels of plant hormones indicate that they have an essential role in the response of plants to infection and colonization with endophytic fungi [52,69]. The infection of endophyte *Epichloë gansuensis* can promote the host endogenous hormones content such as indole-3-acetic acid (IAA), cytokinin (CTK), gibberellin (GA), jasmonic acid (JA), salicylic acid (SA), and the expression of genes, which can alleviate the harm of abiotic stresses on the growth of host plants [70]. Meanwhile, plant hormones, such as IAA, GA, CTK, abscisic acid (ABA), SA and JA function as essential endogenous signal molecules play important role in regulating plant development and response to biotic and abiotic stresses in plants [71,72]. Phenylpropanoid metabolism is enhanced under the regulation of diverse phytohormone signal pathways, such as IAA, JA, GA, and ethylene (ET). So phytohormone can increase lignin deposition by activating the expression of PAL, cinnamate 4-hydroxylase (C_4_H), 4CL, caffeoyl-CoA O-methyltransferase (CCoAOMT), CAD [73]. Our results also revealed that the infection and colonization of endophytic strain ND35 increased host endogenous hormones content such as IAA, GA, zeatin (ZT), SA, and JA and up-regulation of genes expressions related to those hormones, and the endogenous hormone changes may increase lignin content and the activity of key biosynthetic enzymes, such as PAL, 4CL, CAD and POD. These results showed that strain ND35 activated host plant defense signaling pathways and phytohormone signal pathways in the process of establishing a symbiotic system with host plants, to promote plant growth and enhance host plant resistance to biotic and abiotic stress.

## 5. Conclusions

This study revealed that interaction between the cucumber and endophytic *Chaetomium globosum* strain ND35 can contribute significantly to plant growth. The strain ND35 can colonize cortical cells of cucumber and form a symbiotic structure with the host, similar to the periarbuscular membrane and the changes of cell structure, endogenous phytohormones and metabolites primed by the interaction with strain ND35 mediated plant growth. Overall, the infection and colonization events of *C. globosum* strain ND35 increased the cucumber growth through complex regulation of plant hormones biosynthesis and metabolism. Future research on the molecular mechanism of cucumber response to strain ND35 inoculation needs to be conducted for a better understanding of the plant-endophytic fungi interactions.

## Figures and Tables

**Table 1:** Effect of *C. globosum* strain ND35 on the seed germination and seedling growth of cucumber.

Treatment	Spore Concentrations of Strain ND35 (Spores/mL)	Seed Germination Stage			Seedling Stage			
		Radicle Length (mm)	Fresh Weight of Radicle (g)	Dry Weight of Radicle (g)	Plant Height(cm)	Root Length (cm)	Shoot dry Weight (g)	Root Dry Weight (g)
CK	0	18.31 ± 4.68 c	0.108 ± 0.025 c	0.076 ± 0.012 d	16.46 ± 2.47 cd	8.49 ± 1.28 a	0.42 ± 0.045 bc	0.047 ± 0.014 b
T1	1 × 10^4^	19.92 ± 6.84 c	0.113 ± 0.009 c	0.094 ± 0.004 c	17.31 ± 1.46 cd	7.15 ± 1.34 c	0.41 ± 0.043 bc	0.057 ± 0.006 b
T2	1 × 10^5^	25.41 ± 5.49 b	0.402 ± 0.009 b	0.154 ± 0.004 b	19.36 ± 1.96 a	6.64 ± 0.63 c	0.47 ± 0.036 ab	0.060 ± 0.018 ab
T3	1 × 10^6^	30.56 ± 3.57 a	0.593 ± 0.039 a	0.258 ± 0.007 a	18.78 ± 1.38 ab	8.77 ± 1.40 a	0.49 ± 0.036 a	0.079 ± 0.008 a
T4	1 × 10^7^	16.51 ± 3.23 c	0.089 ± 0.009 c	0.057 ± 0.008 e	15.94 ± 1.63 d	8.68 ± 1.11 a	0.48 ± 0.033 a	0.066 ± 0.004 ab
T5	1 × 10^8^	17.79 ± 7.76 c	0.076 ± 0.017 c	0.051 ± 0.007 e	17.69 ± 1.46 bc	8.19 ± 1.52 ab	0.36 ± 0.030 cd	0.060 ± 0.010 ab

CK: The cucumber seeds treated with sterile water. The data in the table are mean ± SD. The different lowercase letters in the same column indicate significant differences (*p* ˂ 0.05).

## Data Availability

The study did not report any data. All other data are available from the corresponding author upon reasonable request.

## Supplementary Materials

The following supporting information can be downloaded at: https://www.mdpi.com/article/10.3390/jof8020180/s1, Figure S1: The detection of biotin content and activity in fermentation liquid of *C. globosum* strain ND35 with high performance liquid chromatography (HPLC) and plant growth test. A: indolebutyric acid (IBA); B: indole-3-acetic acid (IAA); C: gibberellin (GA); D: zeatin (ZT); E: ergosterol; F: The effect of standard solution and strain ND35 crude product of GA and ergosterol on the plant height (F), root length (G), fresh weight (H) and dry weight (I) of cucumber seedlings. GA-S: Standard sample of GA; GA-T: crude product of GA extracted from strain ND35 fermentation broth; Er-S: Standard sample of ergosterol; Er-T: rude product of ergosterol extracted from strain ND35 fermentation broth.; Figure S2: Correlation analysis of patterns of gene expression in each group; Figure S3: The classification and functional enrichment of DEGs based on GO term and KEGG pathway in comparison group CK24 vs. ND24; Figure S4: Validation of the relative expressions of phenylpropanoid biosynthesis and plant hormone signal transduction transcripts by qRT-PCR. Table S1: List of primers used in this study; Table S2: Quantitative determination of GA, ergosterol, ZT, IBA and IAA in the fermentation broth of strain ND35; Table S3: Summary of RNA-seq data; Table S4: Statistical analysis of differentially expressed genes (DEGs); Table S5: Enrichment analysis of Gene Ontology (GO) terms among differentially expressed genes; Table S6: Enrichment of KEGG pathways among differentially expressed genes; Table S7: The expression of genes related to pectin.
