# Supplementary material for: Mechanisms in Growth-Promoting of Cucumber by the Endophytic Fungus Chaetomium globosum Strain ND35
Source: J Fungi (Basel). 2022 Feb 11;8(2):180. doi: 10.3390/jof8020180 (PMC8878499; doi:10.3390/jof8020180)
Supplement: Supplementary file 1 [file jof-08-00180-s001.zip › Supplementary S1.pdf]

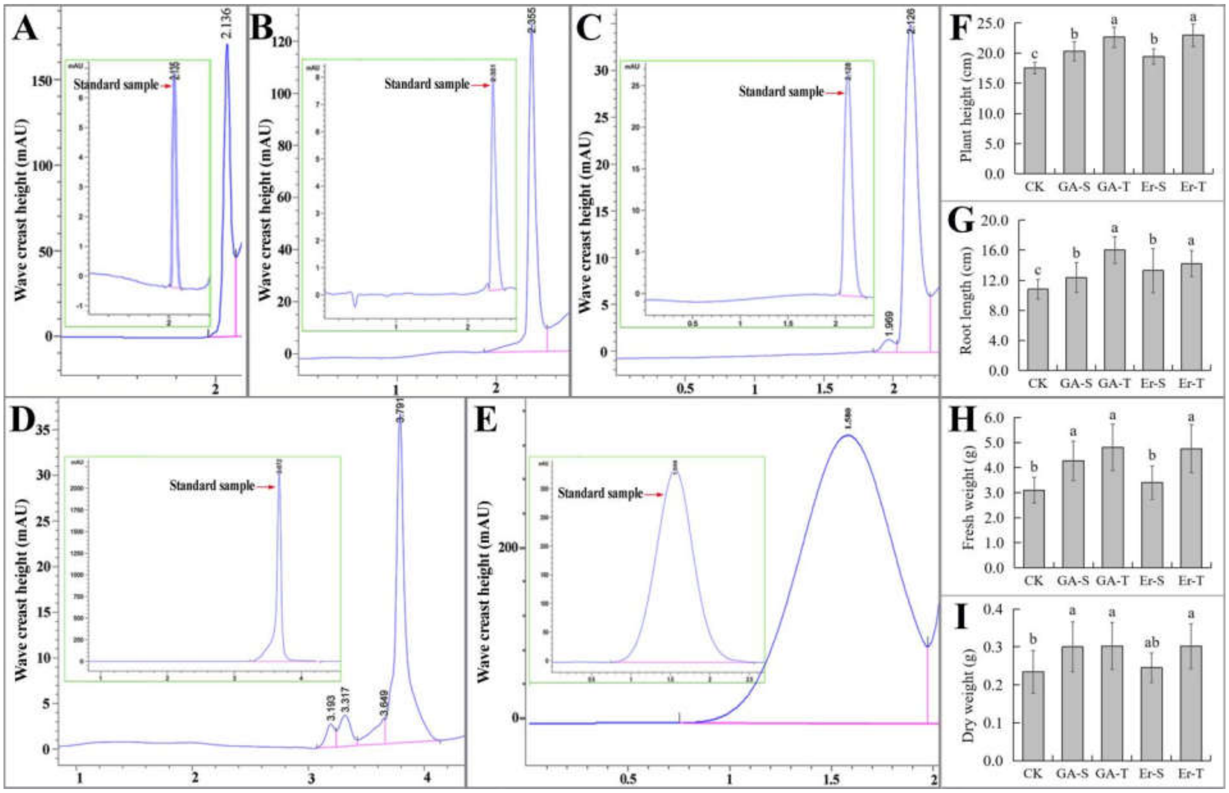

Figure S1. The detection of biotin content and activity in fermentation liquid of *C. globosum* strain ND35 with high performance liquid chromatography (HPLC) and plant growth test.

A: indolebutyric acid (IBA); B:indole-3-acetic acid (IAA); C: gibberellin (GA); D: zeatin (ZT); E: ergosterol; F: The effect of standard solution and strain ND35 crude product of GA and ergosterol on the plant height (F), root length (G), fresh weight (H) and dry weight (I) of cucumber seedlings. GA-S: Standard sample of GA; GA-T: crude product of GA extracted from strain ND35 fermentation broth; Er-S: Standard sample of ergosterol; Er-T: rude product of ergosterol extracted from strain ND35 fermentation broth.

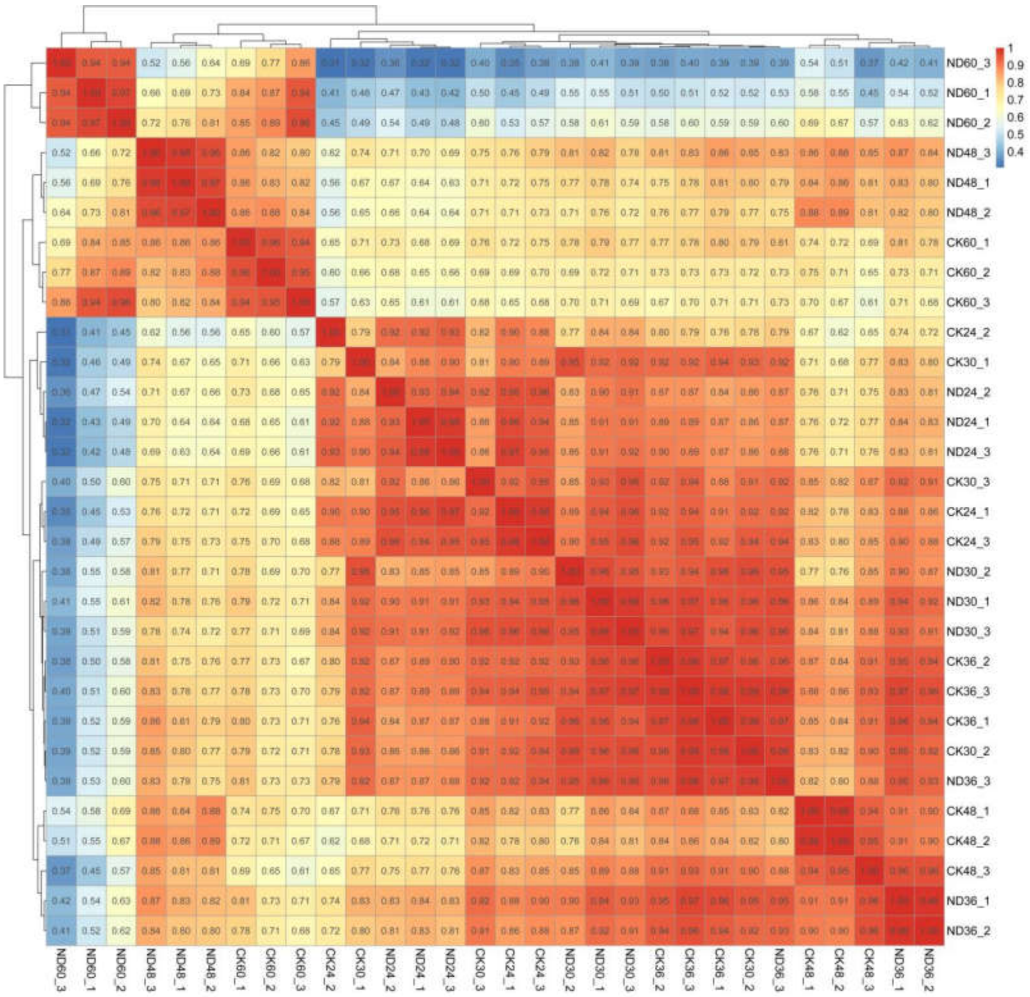

Figure S2. Correlation analysis of patterns of gene expression in each group.

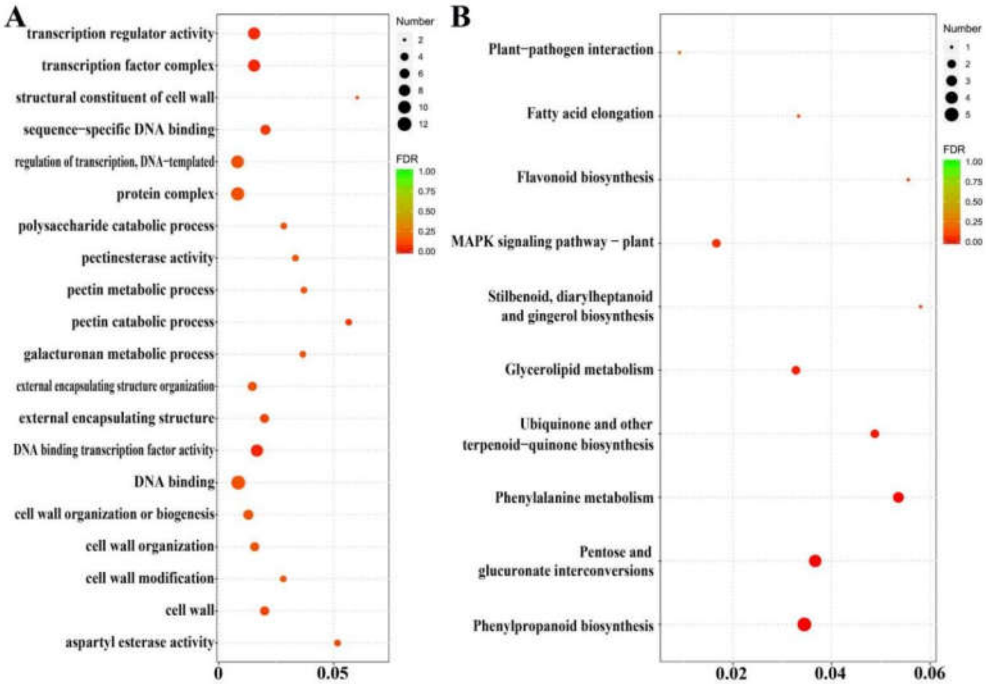

Figure S3. (A,B) The classification and functional enrichment of DEGs based on GO term and KEGG pathway in comparison group CK24 vs. ND24.

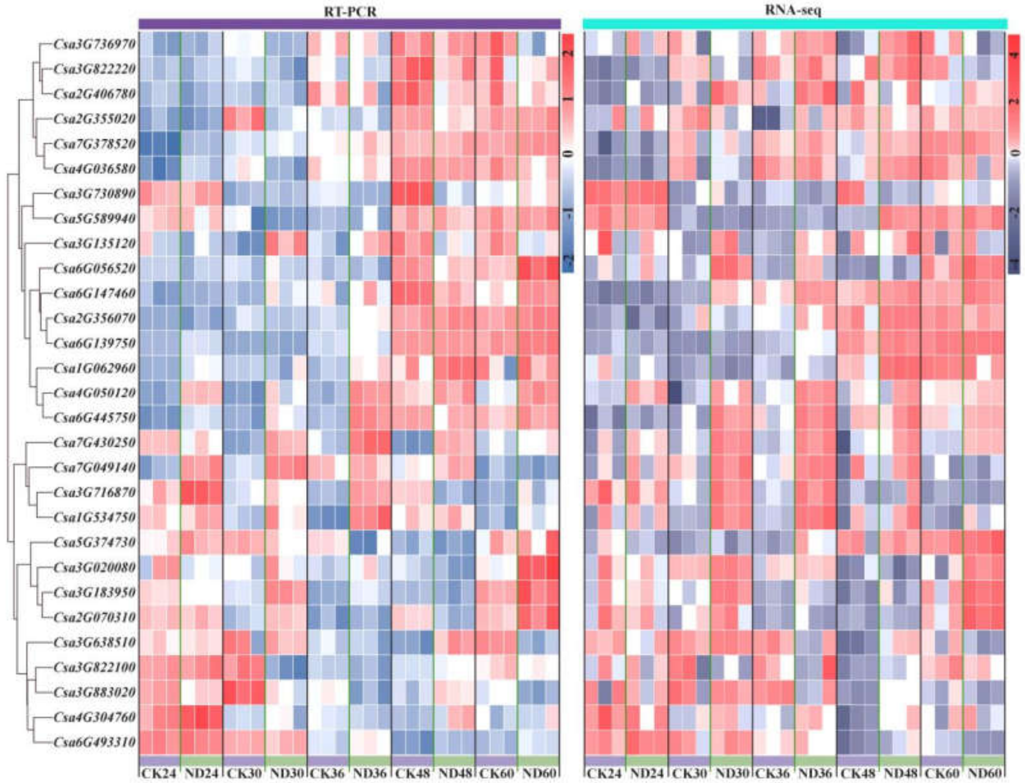

Figure S4. Validation of the relative expressions of phenylpropanoid biosynthesis and plant hormone signal transduction transcripts by qRT-PCR.
